# Relationship of Oral Tumor Thickness with the rate of lymph node metastasis in Neck based on CT Scan

**DOI:** 10.12669/pjms.332.11429

**Published:** 2017

**Authors:** Sohail Ahmed khan, Sadaf Zia, Syeda Uzma Naqvi, Hatem Adel, Syed Omair Adil, Munawar Hussain

**Affiliations:** 1 Dr. Sohail Ahmed Khan, FCPS. Assistant Professor, Dow Institute of Radiology, Dow University of Health Sciences, Karachi, Pakistan; 2 Dr. Sadaf Zia, FCPS. Assistant Professor, Department of E.N.T, Dow International Medical College (Ojha campus), Dow University of Health Sciences, Karachi, Pakistan; 3 Syeda Uzma Naqvi, FCPS. Assistant Professor, Department of E.N.T, Dow International Medical College (Ojha campus), Dow University of Health Sciences, Karachi, Pakistan; 4 Hatem Adel, MBBS. Resident, Dow Institute of Radiology, Dow University of Health Sciences, Karachi, Pakistan; 5 Syed Omair Adil, MS. Research Associate, Dow Institute of Radiology, Dow University of Health Sciences, Karachi, Pakistan; 6 Munawar Hussain, FCPS. Associate Professor, Dow Institute of Radiology, Dow University of Health Sciences, Karachi, Pakistan

**Keywords:** Oral Tumor thickness, Neck metastasis, Neck dissection, CT Scan Neck

## Abstract

**Objective:**

To determine the relationship of tumor thickness of oral lesions with metastasis in neck based on CT scan.

**Methods:**

A total of 58 oral squamous cell carcinoma patients having the median age of 46 (39-55) years. with either gender presented with malignant tumor of buccal mucosa and tongue were prospectively enrolled. A CT Scan with contrast was performed on all patients. Correlation of tumor thickness level with metastasis in neck was calculated using spearman’s rank correlation coefficient test.

**Results:**

Median age of the patients was 46 (39-55) years with preponderance of male gender, i.e. 48 (82.8%). Strong positive significant correlation was observed in between transverse dimension (TS) tumor size and stages of tumor (rho 0.673, p-value <0.001), Anterioposterior (AP) tumor size and stages of tumor (rho 0.675, p-value <0.001), and Craniocaudal (CC) tumor size and stages of tumor (rho 0.771, p-value <0.001).

**Conclusion:**

CT scan of neck with contrast can be used for predicting the positive presence of lymph node in neck with primary tumors having a size of more than 4 mm.

## INTRODUCTION

The presence of neck metastasis in relationship to oral tumors is well known. It is also well documented that presence of neck metastasis reduces the survival rate of the individuals.^1^-^3^ Since early times, clinicians have been trying to find better methods of identifying and predicting the possibility of presence of metastasis in neck, for head and neck cancers.^4^,^5^ The CT scan and MRI are important technical developments/advancements, helping in determining the presence, extent and local/ regional invasion of tumors.^6^-^8^ Investigators also explored the use of Gamma probes for determining occult metastasis in neck with limited success.^9^,^10^

Recent studies are also looking at the relationship of tumor thickness with the presence of lymph node metastasis in neck based on histopathological examination. A meta-analysis reported 0.4 mm as cutoff value for oral SCC.^11^ The oral tumors presenting with occult neck metastasis in early SCC (T1 or T2) may be up to 27-40% which may not be evident either clinically or radiologically.^12^,^13^ Thus necessitating elective neck dissection in these individuals, some of these surgeries may be unnecessary exposing them to the morbidity related to neck dissection.

Radiological investigation of CT scan neck with contrast is used widely thought out the world as a reliable tool for evaluating the presence of lymph nodes in neck; but its relation to the thickness of oral tumor has not been investigated in literature up till now. It also gives the opportunity for 3D analysis of tumors.^14^,^15^ Some investigators also used Ultrasound/MRI for determining the tumor volume.^16^

This study will be important for our study population as oral SCC is becoming increasingly common in our population. The foreign population has a different spectrum of disease; they also have a tendency to present late for medical care. In Pakistan to the best of our knowledge this is perhaps the first study looking at this aspect of oral SCC. For the same reason, this study was conducted with aim to find a non-invasive, reliable and cost-effective method for determining the metastatic potential of lymph nodes in neck in relation to the thickness of oral tumors.

## METHODS

### Data collection procedure

A prospective study was conducted among 58 patients with oral tumor after getting approval from Institutional Review Board (IRB) of Dow University of Health Science (DUHS) from November 2015 to May 2016. Informed consent was also taken from all study participants after explaining the pros and cons of the study. The inclusion criteria were; (1) age more than 18 years, (2) Either gender, (3) having diagnosed Oral SCC (4) Malignant tumor of buccal mucosa and tongue. The exclusion criteria were allergic patients with IV contrast and pregnant ladies.

**Fig.1:**
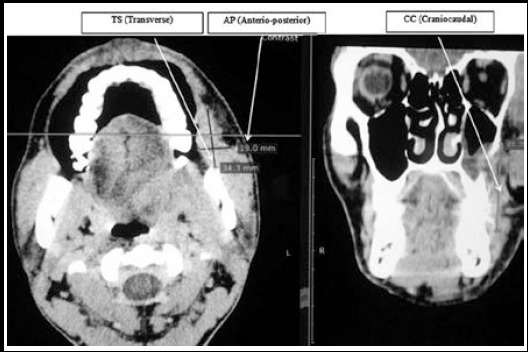
Dimensions of TS, AP and CC as marked on CT Scan Neck with contrast.

CT scan with contrast on 16 slice CT SCAN machines by Siemens was performed on all patients. Measurements of AP (Anterio-Posterior), TS (Transverse), CC(Cranio-caudal) were taken Fig.1. All information including demographics, site of SCC of oral cancer i.e. cheek/buccal mucosa, tongue, floor of mouth, tumor thickness, stage of tumor, involvement of cervical lymph nodes, its size and level measurement (1-7) were noted. Staging of tumors was done according to AJCC guidelines.

### Statistical analysis

SPSS v.20 was used for statistical analysis. Descriptive analysis was explored using median and interquartile range (IQR) for age and tumor thickness level of anterioposterior (AP), transverse dimension (TS) and craniocaudal (CC). Frequency and percentages were calculated for gender, side and site of SCC oral cavity, tongue, floor of mouth and stage of tumor. Association of tumor thickness level of AP, TS and CC with positive neck levels were explored using chi-square test taking <0.05 of level of significance. Correlation of tumor thickness and level of measurements were explored using Spearman’s correlation tests (rho). Significant value for rho was taken as <0.001.

## RESULTS

### Baseline characteristics

Out of total 58 patients with SCC of oral cancer, majority 48 (82.8%) were males while 10 (17.2%) were females. The median age was 46 (39-55) years. There were 34 (58.6%) patients with SCC of right side oral cavity whereas 24 (41.4%) with SCC of left side oral cavity. Cheek/buccal/mucosa was affected in 26 (44.8%) patients. Lateral tongue involvement was observed in 27 (46.6%) while dorsum tongue involvement was observed in 32 (55.2%) patients. Floor of the mouth and posterior tongue was affected in 3 (5.2%) and 2 (3.4%) patients respectively Baseline characteristics of the patients is shown in Table-I.

**Table-I:** Baseline characteristics of the patients (n=58).

	*n*	*%*
Age, years	46 (39-55)^†^	
***Gender***		
Male	48	82.8
Female	10	17.2
Side of SCC Oral Cavity		
Right	34	58.6
Left	24	41.4
***Cheek/Buccal/mucosa***		
Yes	26	44.8
No	32	55.2
Tongue Involvement		
Lateral Margin	27	46.6
Dorsum	4	6.9
***Floor of mouth***		
Yes	3	5.2
No	55	94.8
Post Tongue		
Yes	2	3.4
No	56	96.6

† median(IQR)

### Stages of tumor

Tumor stage 2 was found to be higher 23 (39.7%) followed by tumor stage 3 in 17 (29.3%), stage 4 in 11 (19%) while stage 1 tumor was found in 7 (12.1%) patients.

### Tumor Thickness

**Fig.2:**
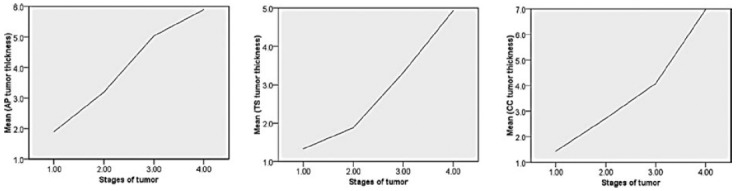
Comparison of mean tumor thickness level with stages of tumor.

Median AP, TS and CC tumor thickness was 3.7 (2.67-5.92) mm, 2.6 (1.3-3.7) mm and 3.7 (2.4-5.92) mm respectively. Fig.2 shows the comparison of tumor thickness level with stages of tumor.

### Correlation among level of Neck node measurements and tumor thickness

Strong negative correlation was observed in between AP tumor thickness and level 1 measurement (rho -0.702, p-value 0.12) whereas moderate negative correlation was observed among TS tumor thickness and level 1 measurement (rho -0.612, p-value 0.19) and CC tumor thickness and level 1 measurement (rho -0.40, p-value 0.60).

Moderate positive correlation was observed in between AP tumor thickness and level 2 measurement (rho 0.43, p-value 0.33), strong positive correlation was observed among TS tumor thickness and level 2 measurement (rho 0.709, p-value 0.07), whereas weak positive correlation was observed in between CC tumor thickness and level 2 measurements (rho 0.103, p-value 0.87).

### Correlation among stages of tumor and tumor thickness

Strong positive correlation was observed in between AP tumor size and stages of tumor (rho 0.675, p-value <0.001), TS tumor size and stages of tumor (rho 0.673, p-value <0.001) and CC tumor size and stages of tumor (rho 0.771, p-value <0.001).

### Tumor thickness and positive lymph nodes

Tumor thickness level of AP, TS and CC was insignificantly higher among patients with level 1 lymph node involvement as compared to the patients with level 2 lymph node involvement (p-value >0.05) (Table-II).

**Table-II:** Comparison of tumor thickness with positive neck levels (n=58).

*Level of lymph node involved*	*Anterioposterior Tumor Thickness*		*p-value ^‡^*	*Transverse Dimension Tumor Thickness*		*p-value ^‡^*	*Cranio Caudal Tumor Thickness*		*p-value ^‡^*

	*Non-Metastasis n (%)*	*Metastasis n (%)*	*n (%)*	*Non-Metastasis n (%)*	*Metastasis n (%)*	*n (%)*	*Non-Metastasis n (%)*	*Metastasis n (%)*
1	17 (60.7)	11 (39.3)	0.907	22 (78.6)	6 (21.4)	0.832	11 (50)	11 (50)	0.446
2	10 (62.5)	6 (37.5)		13 (81.2)	3 (18.8)		7 (70)	3 (30)	

‡ Chi-square test applied taking p-value significant at <0.05 <4 tumor thickness level is labeled as non-metastasis, ≥4 tumor thickness level is labeled as metastasis.

## DISCUSSION

Our study showed a strong positive significant correlation in between the tumors sizes of TS, AP and CC with the radiological staging of the tumor. There was no study which exactly matches the present study but we found a study based on MRI comparing the volumes of the tumor to lymph node metastasis^14^. In our study a strong positive correlation was also observed among TS tumor thickness and level 2 lymph nodes. Thus indicating that for primary tumors of more than 4mm TS, the lymph nodes have a positive correlation for being positive for tumor. Madana J et al compared the CT measurements of Tumor thickness with histology and found them to closely relate to each other.^17^ Park JO found diagnostic accuracy with MRI and histopathological specimens in predicting the tumors size.^18^ However the availability and the patient tolerance of CT scan is better than an MRI Scan. Our population having SCC is mostly coming from lower socio-economic class. Therefore, CT Scan would be a better option for this class of patients.

The lymph nodes which are suspected to be metastatic bear the features of being rounded and more than 1cm in size. Lodder WL. et al in their study found a positive correlation with a tumor size of 7 mm as predictive of positive lymph nodes using an ultrasound probe intra-orally.^16^ However, Lwin CT et al had a conflicting result with MRI evaluation.^19^

The findings of our study also showed preponderance of right side oral SCC. This could be due to the reason that most people are right handed so they have a tendency for keeping pan/ betel nut on right side of oral cavity. Moreover, male gender were predominantly higher as compared to females which is similar to the previously published worldwide report.^20^ In addition to this, as reported by previous studies, tongue tumors was also found higher in the lateral wall of tongue in our study.^20^

In relation to SCC of oral cavity, it is well documented that the presence of metastatic neck lymph node decreases the survival of cancer patients.^3^ It is thought that the elective neck dissection increases the disease free survival and overall wellbeing of cancer patients.^3^,^11^ It raises an important question about the affectivity of elective neck dissection.^20^ It is reported that most of the elective neck dissection may be unnecessary but overall it has better prognostic value when compared to wait and watch policy.^11^,^20^-^23^ Thus, increasing the morbidity of these patients undergoing elective neck dissection for complications such as shoulder joint movement problems, neck heamatoma and infections.^14^

As seen with various studies on histopathological examination, the thickness of primary tumor can be taken as a risk factor for developing lymph node metastasis in neck.^11^,^24^,^25^ O’Brien C J proposed a depth of 4mm of tumor thickness to correlate significantly with nodal metastasis even in T1-T2 oral tumors based on histopathology.^26^ Due to the limited availability of PET Scan and also the high cost of the investigation, we need to look for ways to detect cancer in neck as early as possible without putting the burden on our population economically, as this is a disease of the lower socio-economic group in our country. CT scan is the cost effective and reliable modality of investigation which is already available all over the country and it is internationally accepted as well.

### Limitations

Further studies are recommended taking histopathology as gold standard which not only validate the CT Scan being used for the evaluation of neck disease but will also give subsequent advice of elective neck dissection based on tumor thickness measurements.

## CONCLUSION

CT Scan of neck with contrast can be used as a non-invasive tool for predicting the positive presence of metastatic lymph node in neck with primary tumors having a size of more than 4 mm. Elective neck dissection is justified using the criteria of primary tumor thickness of more than 4mm based on CT Scan measurements which is a low cost and easily available option for patients with SCC of oral cavity.
